# Dr. W. A. Hammond

**Published:** 1878-05

**Authors:** 


DR. W. A. HAMMOND.—The bill to reinstate Dr. Hammond as Surgeon-General and place him upon the retired list has passed the Senate and the House of Representatives. It now merely awaits the approval of the President.

